# High-Pass Filtering of Input Signals by the I_h_ Current in a Non-Spiking Neuron, the Retinal Rod Bipolar Cell

**DOI:** 10.1371/journal.pone.0001327

**Published:** 2007-12-19

**Authors:** Lorenzo Cangiano, Claudia Gargini, Luca Della Santina, Gian Carlo Demontis, Luigi Cervetto

**Affiliations:** 1 Dipartimento di Psichiatria e Neurobiologia, Università di Pisa, Pisa, Italy; 2 Dipartimento di Fisiologia Umana “G. Moruzzi”, Università di Pisa, Pisa, Italy; Emory University, United States of America

## Abstract

Hyperpolarization–activated cyclic nucleotide–sensitive (HCN) channels mediate the I_f_ current in heart and I_h_ throughout the nervous system. In spiking neurons I_h_ participates primarily in different forms of rhythmic activity. Little is known, however, about its role in neurons operating with graded potentials as in the retina, where all four channel isoforms are expressed. Intriguing evidence for an involvement of I_h_ in early visual processing are the side effects reported, in dim light or darkness, by cardiac patients treated with HCN inhibitors. Moreover, electroretinographic recordings indicate that these drugs affect temporal processing in the outer retina. Here we analyzed the functional role of HCN channels in rod bipolar cells (RBCs) of the mouse. Perforated–patch recordings in the dark–adapted slice found that RBCs exhibit I_h_, and that this is sensitive to the specific blocker ZD7288. RBC input impedance, explored by sinusoidal frequency–modulated current stimuli (0.1–30 Hz), displays band–pass behavior in the range of I_h_ activation. Theoretical modeling and pharmacological blockade demonstrate that high–pass filtering of input signals by I_h_, in combination with low–pass filtering by passive properties, fully accounts for this frequency–tuning. Correcting for the depolarization introduced by shunting through the pipette–membrane seal, leads to predict that in darkness I_h_ is tonically active in RBCs and quickens their responses to dim light stimuli. Immunohistochemistry targeting candidate subunit isoforms HCN1–2, in combination with markers of RBCs (PKC) and rod–RBC synaptic contacts (bassoon, mGluR6, Kv1.3), suggests that RBCs express HCN2 on the tip of their dendrites. The functional properties conferred by I_h_ onto RBCs may contribute to shape the retina's light response and explain the visual side effects of HCN inhibitors.

## Introduction

A hyperpolarization–activated current (I_h_) with properties similar to cardiac funny current (I_f_, reviewed by [1]) is widely distributed in the brain, as well as in sensory systems (see [2]). The I_h_–carrying channel has been identified as a hyperpolarization–activated cyclic nucleotide–sensitive channel (HCN), a member of the voltage–gated K^+^ channel family that is closely related to the cyclic nucleotide–gated channels [3]. Molecularly, mammalian HCN are assembled as homo– or hetero–tetramers from protein subunits encoded by the four different genes HCN1–4 [4], [5], [6]. HCN open in response to membrane hyperpolarization and close upon depolarization but do not inactivate, a property that enables them to contribute as a standing current to neuronal excitability [7], [8]. Cytosolic cAMP shifts their range of activation to more depolarized potentials [9] (but see [10]). Their kinetics of activation and deactivation is slow, with time constants up to hundreds of milliseconds or more. Being the HCN permeable to both Na^+^ and K^+^, they normally carry an inward (i.e. depolarizing) current, driving a neuron's membrane potential away from further HCN activation. The HCN can thus operate as a slow negative–feedback mechanism.

The HCN have been found responsible for a variety of physiological functions including control of pacemaker activity [1], [11], [12] and regulation of synaptic integration in neuronal dendrites [13]. In the retina, pharmacological blockade of I_h_ has been shown to interfere with the temporal processing of visual signals [14], [15]. Furthermore, visual disturbances, mainly phosphenes, occur in cardiac patients treated with I_f_ inhibitors (reviewed by [16]). Despite the wealth of morphological and electrophysiological data showing a diffuse distribution of HCN in retinal neurons [17]–[22], only a few studies address the role of I_h_ in processing visual information. Specifically, HCN gating in the rod inner segment has been shown to accelerate the kinetics of large voltage responses well beyond the intrinsic limits set by the phototransductive machinery [23]–[25].

Here we characterized the functional role of HCN channels in second order neurons of the rod pathway, the rod bipolar cells (RBCs), in dark–adapted mice. Individually recorded RBCs display I_h_, possibly attributable to HCN2 channels that immunolabeling suggests to be expressed at their dendritic tips. In darkness this current is predicted to endow RBCs with frequency–tuning, thus sharpening the time course of light responses starting from the range of single photon absorption signaling.

## Materials and Methods

### Electrophysiology

**Figure 1:**
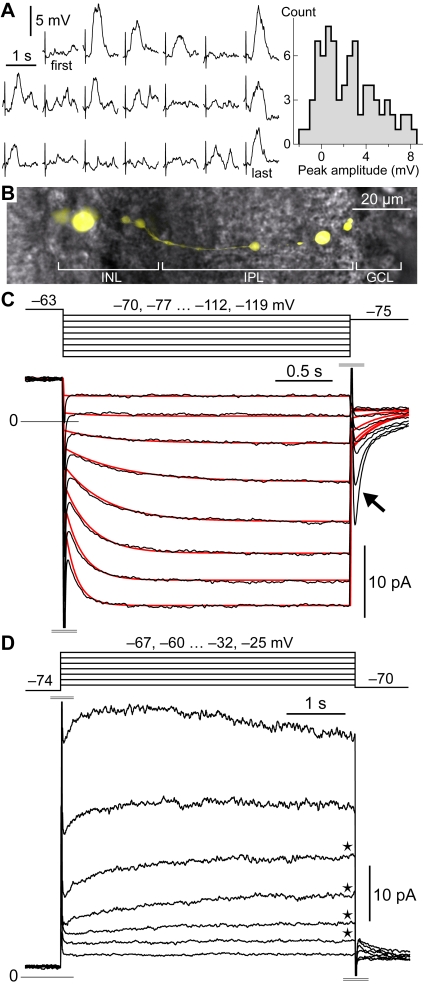
Individually recorded RBCs display an I_h_–like current. A, Perforated–patch clamp recording in the retina slice of an RBC. Dim flashes of green light delivered at the cell's response threshold evoke highly variable depolarizing potentials in current–clamp (constant flash intensity, 6 ms flash duration, 4 s inter–flash interval, 20 consecutive episodes). Evidence for a quantal nature of these responses can be seen in the distribution of their peak amplitudes (histogram shows binned data from 79 flash episodes). B, The same RBC stained with LY at the end of the experiment. C, Voltage–clamp protocol applied to this RBC shows the progressive activation of a slow inward I_h_–like current in response to increasingly hyperpolarizing voltage steps, from a holding potential of −63 mV (black traces). The model of *eqs. 1*–*2* (red traces) provides a very good fit of the experimental records with parameters *G_h_* = 0.144 nS, *V_0.5_* = −89.5 mV, *k_m_* = 5.0 mV, *tau_max_* = 510 ms; *G_leak_* = 0.340 nS. D, In the same neuron, a voltage–clamp protocol of increasingly depolarizing steps from a holding potential of −74 mV, reveals an outward current with slow activation time course (traces with stars). At potentials above about −40 mV a larger current appears, with fast activation and slow inactivation kinetics. Experimental traces in C and D are averages of 11 and 10 trials, respectively. Stimulation artifacts are trimmed.

Mice (C57Bl6/J) in the age range P26–170 were dark–adapted for 1–2 hours, anesthetized by i.p. injection of 2,2,2–tribromethanol (Sigma–Aldrich, St. Louis MO; 15 mg/kg), and their retinae rapidly extracted through a corneal incision into cooled saline under dim red light. Each retina was laid vitreal side down on filter paper, embedded in a thin layer of low–gelling temperature agarose (Sigma–Aldrich) and sliced in 250 µm sections with a manual tissue chopper mod. 600 (The Vibratome Company, St. Louis MO). Slices were secured within the recording chamber with a nylon net, continuously perfused with O_2_/CO_2_–bubbled AMES medium (Sigma–Aldrich) and visualized in infrared under an upright microscope (Leica Microsystems, Wetzlar Germany). Most of the experiments were done near room temperature (∼23°C), which allowed long–lasting stable recordings. Unless otherwise stated, data presented below were collected at this temperature. Control measurements near physiological temperatures (∼35°C) were obtained in a limited number of cells. Pipettes for perforated patch recording were pulled with a P–97 (Sutter Instrument, Novato CA) and filled with a solution containing in mM 94 K_2_SO_4_, 20 KCl, 10 NaCl, 5 Pipes, corrected to a pH of 7.20. The back–filling solution also contained 0.5 mg/ml Lucifer Yellow (LY) and 0.2 mg/ml Amphotericin–B (both from Sigma–Aldrich), the latter pre–dissolved in DMSO at 30 mg/ml. Pipettes (6–9 MΩ) were advanced in the external third of the inner nuclear layer to a significant depth from the slice surface. A giga–seal was formed, followed shortly after by the development of low–resistance access via patch perforation (69±36 MΩ). Müller glia were identified by their input resistance, an order of magnitude smaller than that of bipolar cells (128±48 MΩ versus 3.3±1.0 GΩ, p<0.001 Wilcoxon–Mann–Whitney test), and discarded. Neurons were stained with LY at the end of the experiment by rupturing the patch. Fluorescence images acquired on different focus planes were blended in Photoshop CS2 to reconstruct cell morphology. Rod bipolar cells (RBCs) were distinguished by their characteristic globular axon terminals and their level of stratification in the inner plexiform layer [26]. Recordings were made with an Axopatch 1D amplifier with its low–pass filter set at 500 Hz, digitized at 5 kHz and acquired by pClamp 8 software (both from Axon Instruments, Foster City CA). Stray capacitance was minimized with a glass cover slip treated with Sigmacote (Sigma–Aldrich), placed on the liquid surface of the chamber just behind the pipette tip. As in other work the perforated–patch technique led to stable I_h_ currents for the entire duration of our recordings, lasting on average more than one hour. The I_h_ activation function could not be reliably estimated by tail current analysis as, upon repolarization from very negative potentials, a transient inward current with inactivation kinetics overlapping the time course of I_h_ deactivation was often present (see Results). We circumvented the problem by fitting the family of current trajectories *during* the hyperpolarizing step potentials, with the sum of three terms: an ohmic leakage, an I_h_ steady–state, and an I_h_ transitory component having mono–exponential kinetics:
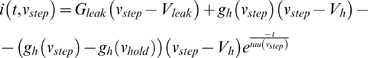
(1)with the steady–state I_h_ conductance at potential *v*, given by a Boltzmann function,
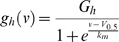
(2)
*G_leak_* is the voltage– and time–independent leakage conductance, having reversal potential *V_leak_*. *G_h_* is the I_h_ maximum conductance, *V_0.5_* its half–activation potential, *k_m_* the inverse slope factor, and *tau(v_step_)* its voltage–dependent gating time constant. The I_h_ reversal potential *V_h_* was separately calculated to be −34 mV using the Goldman–Hodgkin–Katz equation and assuming a Na^+^/K^+^ permeability ratio of the HCN channel of 0.33 [27], while all other parameters were obtained as the set that best fit *eq. 1* to the experimental data (very good fitting occurred for step potentials more negative than about −70 mV; Figure 1C). These same parameter values were also used in building the simplified cellular model described below. This included single exponential kinetics for I_h_, which, while keeping the model simple, was sufficient to predict quite well the quantitative aspects of band–bass behavior in RBCs. In a number of experiments ZD 7288 (Tocris, Bristol United Kingdom) was added to the saline to block I_h_. Membrane potentials were corrected for a liquid junctional potential calculated to be 10 mV (JPCalc, Axon Instruments). Dim flashes of green light of duration 0.2–18 ms were delivered to the preparation by an LED (OD520; Optodiode Corp., Newbury Park CA), through an optical band–pass filter (509–519 nm) and a neutral density filter (2 log units), placed beneath the recording chamber. Slices were thus uniformly illuminated. Flash energy was empirically adjusted to the threshold sensitivity of the recorded cell such that, on repetitive stimulation, occasional failures occurred (0.3–1 photons/µm^2^·flash measured at bottom of the chamber). Precise scaling was thereafter obtained by varying flash duration, while maintaining LED power output constant. Except when otherwise stated, data are expressed as mean±s.d.

### Input Impedance Measurement

We explored the neuronal frequency–response characteristics by delivering, in current–clamp, a sinusoidal current stimulus of 50 s duration (*T*), modulated in frequency continuously and monotonically between 0.1 Hz (*ƒ_min_*) and 30 Hz (*ƒ_max_*). Referred to in the literature as a *ZAP* stimulus [28], we modified it in order to give equal representation in the time domain to each frequency decade (i.e. same time spent between 0.1–1 Hz as between 1–10 Hz). This was ensured by varying the sinusoid frequency according to the exponential function
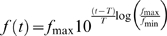
The current stimulus delivered to the neuron was thus
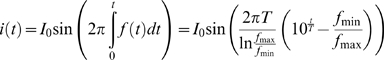
To approach system linearity, a prerequisite for harmonic analysis, we set *I_0_* such that the peak–to–peak amplitude of the voltage response remained in most cases below 10 mV. This precaution also ensured that the measured input–output relation of the neuron was relevant to its physiological response during dim light stimulation. Up to 9 sweeps were averaged in order to increase signal over noise. The cells' complex input impedance at the soma was obtained from the ratio of the voltage response FFT to the current input FFT. The real modulus of the complex impedance (*impedance profile*) was plotted between 0.1 and 30 Hz. This analysis was performed on a personal computer with Axograph 4.9 software (Axon Instruments), using custom written routines.

### Input Impedance Modeling

The role of I_h_ in endowing RBCs with frequency–tuning was explored by using a simplified (or reduced) cellular model of each recorded neuron, consisting of membrane capacitance, ohmic leakage conductance and a single active ionic current represented by I_h_. The model thus did not include either the outward currents (their activation range didn't overlap that of I_h_, see Results), or the inactivating transient inward current (see Results). On the basis of direct experimental tests [29], RBCs were assumed to be well described by a single isopotential compartment. Capacitances were estimated, on a cell–by–cell basis, by fitting with single exponentials their voltage responses to small step currents (not shown). These were delivered in the narrow membrane potential range in which I_h_ and outward currents were not significantly activated (−70 to −75 mV). Leakage and I_h_ conductance parameters were estimated, for each individual RBC, as described above. The simplified model cell can be linearized (in a *small signals* approximation), and its complex impedance derived as a function of frequency and membrane potential [30] and eqs. 18–19 in [31]. The behavior of each model cell was compared to that of its corresponding recorded RBC, by plotting their respective impedance profiles obtained at the same membrane potentials. Note that the parameters describing each model cell were specified *a priori* and not adjusted post–hoc to improve the match between theoretical and experimental impedance profiles (i.e. no free parameters).

### Immunohistochemistry

Adult mice (∼2 months old) were anesthetized by i.p. injection of 2,2,2–tribromethanol (15 mg/kg), eyes were enucleated and immersion–fixed in paraformaldehyde 4% for 15 min, washed in 0.1 M phosphate–buffered saline (PBS; pH 7.4), cryoprotected in 30% sucrose overnight. Eyes were then embedded in Tissue Tek Optimal Cutting Temperature (OCT) compound (Miles Inc., Elkhart IN), frozen at −20°C and serially sectioned at 18 µm on a cryostat. Sections were then collected on gelatin coated slides. Primary antibodies (see also Table 1) were anti–Protein Kinase C (PKC, Sigma–Aldrich; 1∶200), anti–HCN1, anti–HCN2 and anti–Kv1.3 (Alomone, Jerusalem Israel; 1∶200), anti–mGluR6 (Neuromics, Edina MN; 1∶1000), anti–bassoon (Stressgen, San Diego CA; 1∶1000). For all labelings, washes were for 3×5 min in 0.1 M PBS at room temperature, 1% bovine serum albumin (BSA; used for blocking non–specific bindings) and 0.03% Triton ×100 (to induce tissue permeabilization). Primary antibodies were incubated in this last solution at 4°C overnight. Secondary antibodies were anti–mouse or anti–rabbit conjugated with Alexa Fluor 488 (Molecular Probes, Eugene OR; 1∶200) and Alexa Fluor 568 (1∶200) in PBS at room temperature for 2 hours. The ratio of anti–HCN1/2 antibody to its immunizing peptide in pre–incubation controls was of 1∶1 by weight. Retinal preparations were examined with a Leica TCS–NT confocal microscope equipped with a krypton–argon laser. Files were processed with Photoshop CS2 (Adobe Systems, San Jose CA).

**Table 1:** Primary antibody information.

Target	Source	Catalog (Lot) #	Host	Type	Immunogen	Recognized Bands(a)
PKC	Sigma–A.	P5704 (65K4877)	Mouse	Monoclonal clone MC5	Purified bovine brain PKC	One band 80 kDa
HCN1(b)	Alomone	APC-056 (AN-01, AN-02)	Rabbit	Polyclonal	Peptide (C)KPNSASNSRDDGNSV-YPSK, residues 6–24 of rat HCN1	One band ∼110 kDa
HCN2(b) (c)	Alomone	APC-030 (AN-01, AN-02)	Rabbit	Polyclonal	Peptide (C)EEAGPAGEPRGSQAS, residues 147–161 of human HCN2	Two bands ∼97, ∼55 kDa
Kv1.3	Alomone	APC-101 (AN-02)	Rabbit	Polyclonal	Peptide KDYPASTSQDSFEA(C), residues 211–224 of human Kv1.3	Two bands ∼150, ∼50 kDa
mGluR6	Neuromics	RA13105	Rabbit	Polyclonal	Peptide AAPPQNENAEDAK, c–terminus of rat mGluR6	Two bands around 217 kDa
Bassoon	Stressgen	VAM–PS003 (B303420, B403404)	Mouse	Monoclonal cl. SAP7F407	Recombinant rat bassoon fragment (738–1035) expressed as a GST fusion protein in *E. coli*	One band 400 kDa+proteolytic degradation bands 97–400 kDa

(a) Manufacturer's technical information.

(b) Pre–incubation with the control immunizing peptide abolished immunoblot bands^(a)^, as well as retinal staining (Fig. 8A/B, right panels).

(c) A recent study on reticular thalamic neurons [73] reported that the staining pattern given by this antibody in *wild type* animals, disappeared in an HCN2 knock–out (–/–).

## Results

### Perforated patch–clamp recording of rod bipolar cells

Recordings were obtained, in dark–adapted retinal slices maintained near room temperature (∼23°C), from neurons located in the outer third of the inner nuclear layer (INL) using the perforated–patch technique. Rod bipolar cells (RBCs; *n* = 25) were identified functionally by the polarity and time course of the potentials evoked by dim flashes of light (*n* = 17; example from one cell in Figure 1A), and morphologically by rupturing the patch to stain with LY at the end of the experiment (*n* = 18; Figure 1B). Of 17 light–responding RBCs, 16 showed evidence of quantal amplitude fluctuation at response threshold (Figure 1A). The only other neurons encountered in the outer third of the INL were, occasionally, cone bipolar cells (∼20% of all stained neurons). Overall, RBCs had in darkness a resting membrane potential (V_dark_) of −74.7±4.7 mV, an input resistance of 3.2±1.0 GΩ and a capacitance (*C*) of 25.4±6.7 pF. Recorded values of V_dark_ are likely to be significantly more positive than the true values due to the shunt introduced by patching with a seal resistance in the GΩ range, on a small–sized/high–impedance neuron such as the rod bipolar cell [32]. In a later section we estimate the magnitude of this important bias and conclude that the true V_dark_ of unperturbed RBCs can be expected to be more negative than −80 mV.

### RBCs express a current with the characteristics of I_h_

In order to reveal the presence of I_h_ we voltage–clamped the cells at a holding potential of −55 or −63 mV, and imposed progressively more hyperpolarizing steps of 2.5 s duration in 10 or 7 mV increments. A slow–activating, non–inactivating inward current appeared in all 25 RBCs (Figure 1C, black traces). The current time course was well fitted by a single exponential function with a time constant markedly dependent on step voltage, reaching its peak value (*tau_max_*) of 443±102 ms (*n* = 25) around the half–activation potential. This was the only significant active current we observed in RBCs upon hyperpolarization to potentials more negative than −70 mV, a statement supported by the good fit to the experimental traces of a model membrane with only ohmic leakage and I_h_ (Figure 1C, red traces; *eq. 1* in Materials and Methods) as well as by the experiments using a blocker (see below). The putative I_h_ had a conductance at full activation (*G_h_*) of 0.163±0.076 nS, a half activation potential (*V_0.5_*) of −91.4±4.1 mV and an inverse slope factor (*k_m_*) of 6.3±0.7 mV (*n* = 25). The leakage conductance of RBCs (*G_leak_*) was 0.338±0.170 nS, which is an overestimate due to seal resistance shunt (see below). At the end of the hyperpolarizing steps, cells were returned to a potential of −65 or −75 mV to observe tail currents. In 19 of 25 RBCs a large transient inward current appeared upon repolarization from potentials more negative than −90/−100 mV (Figure 1C, arrow), which obscured the deactivation current predicted by model membrane for I_h_ (Figure 1C, red traces). This current, resistant to an I_h_ specific blocker (see below), is similar to that mediated by T–type Ca^2+^ channels observed in bipolar cells of the rat [21], [33], [34] and shown to participate in synaptic transmission to amacrine cells [35], [36]. A majority of RBCs (*n* = 21) were also examined for currents activated upon depolarization using a protocol of progressively more positive steps (2.5 or 5 s duration) from a holding potential of −85 or −74 mV. Two types of outward current were distinguishable based on activation range and kinetics. The first (*n* = 19), recruited already at −70/−60 mV, had a slower activation kinetics than that of the putative I_h_, but similarly showed no sign of inactivation (Figure 1D, traces with stars). It resembled the I_Kx_ present in rods [24], [37]. The other component (*n* = 21), appearing above −50/−40 mV, activated fast and inactivated slowly (Figure 1D). This second current was rather large—up to hundreds of pA when the RBCs were depolarized above 0 mV (cf. [38]).

### The I_h_–like current is sensitive with high affinity to the specific blocker ZD7288

**Figure 2:**
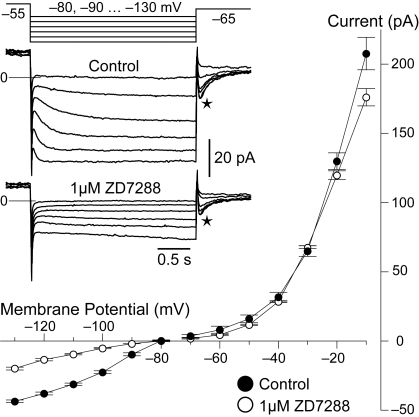
The I_h_ blocker ZD7288 has high affinity and specificity for the hyperpolarization–activated current. Perfusion of the organic drug abolished almost entirely the putative I_h_ current already at a concentration of 1 µM (inset traces, averages of 4, were obtained with the voltage–clamp protocol of hyperpolarizing steps shown at the top). The specificity of its action can be appreciated on the I–V plot, which shows current amplitudes measured at the end of the voltage steps of two different protocols (see also Results and Figure 1), one targeted for inward currents (hyperpolarizing steps) and the other for outward currents (depolarizing steps). After about 25 min (empty circles) ZD7288 strongly reduced the currents activated below −80 mV, compared to control conditions (full circles). The residual current includes an ohmic leakage. Plot displays means and standard errors.

A definitive identification of I_h_ required the use of a well characterized and specific blocker such as ZD7288 [39], [40]. We bath applied the drug at the relatively low concentrations of 5 µM (n = 5) or 1 µM (n = 4) while monitoring its effect on the currents activated by the voltage–clamp protocols described above. A final wash was not attempted because with the organic blockers of I_h_ this is known to require an exceedingly long time [39]. In all cases ZD7288 was effective in abolishing or drastically reducing the presumptive I_h_ current within 10–25 minutes, while leaving outward currents essentially unaffected (Figure 2). The inward current with slow kinetics activated by hyperpolarization in RBCs is thus identified as I_h_. When sufficient time was allowed for a complete blockade of I_h_, the residual current observed during steps to potentials more negative than −70 mV was entirely ohmic (n = 6, not shown). Note that tail currents persisted in ZD7288 (Figure 2, stars) with only a moderate reduction in amplitude due to block of the deactivating I_h_ component, which matched that predicted by fitting experimental traces with eq. 1.

### RBCs exhibit band–pass behavior in current–clamp

**Figure 3:**
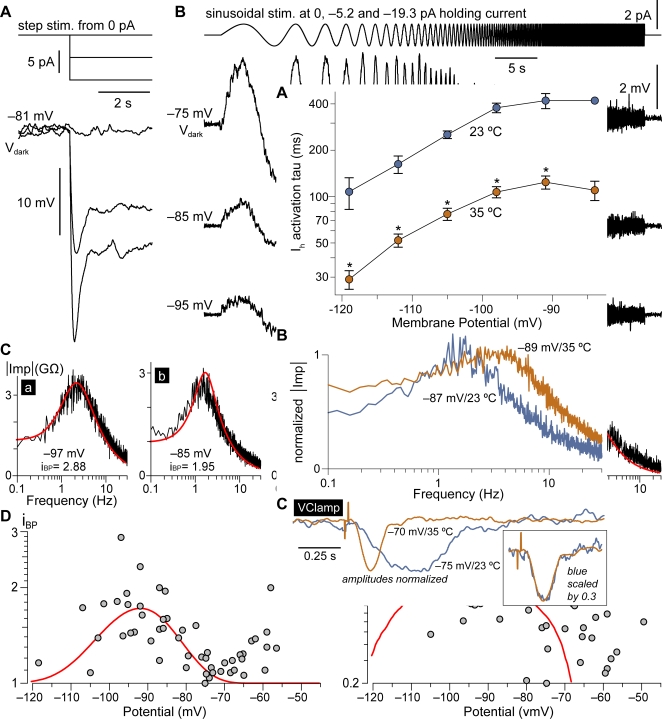
RBCs display band–pass behavior in current–clamp. I_h_, in its range of activation, fully accounts for it. A, RBC subjected to negative current steps from rest displayed voltage overshoots at step onset—a classical hallmark of I_h_. B, The frequency response of another RBC was explored with sinusoidal current stimuli of constant amplitude but modulated in frequency (0.1 to 30 Hz; top trace). When the stimulus was delivered at −75 mV the amplitude of the cell's voltage response decreased progressively with increasing frequency. At two more hyperpolarized potentials, on the other hand, the peak response occurred in the middle of the stimulus, at an intermediate frequency. Traces are averages of 6–9 sweeps. C, Graphs show, as a function of frequency, the modulus of input impedance computed from sinusoidal stimulus–response pairs (noisy black traces; see Materials and Methods). A selection from several RBCs, and covering a range of potentials, is presented in a–e. Band–pass behavior is clear–cut in a–c and in e. The membrane potential and a band–pass index i_BP_ (see Results) are given for each graph. Band–pass impedance profiles at −82 mV and more negative (a–c) are predicted by a simplified theoretical model of each RBC, which includes passive properties and I_h_ as the only active current (see Results). Theoretical impedance profiles (red traces) were derived by linearizing the model (see Materials and Methods) and thus apply to small input signals. D, Summary of data from all RBCs in the form of i_BP_ versus tested potential (circles). Band–pass behavior is expressed <−75 mV and >−70 mV. The average i_BP_ predicted by all RBC theoretical models (red curve) shows that, negative to −75 mV, the observed frequency tuning is fully explained by I_h_, while above −70 mV some other current must come into play. E, Summary of resonant (peak response) frequency data from all RBCs (circles). The models correctly predict, in a wide potential range centered around I_h_ half–activation, an average value of about 1 Hz (red curve).

The impact of I_h_ on the behavior of a neuron is often exemplified by the voltage sag observed in response to hyperpolarizing current steps [2]. This sag, which we also found in all 6 RBCs tested (Figure 3A), follows from I_h_ acting as a slow negative–feedback mechanism, opposing changes in membrane potential both in the hyperpolarizing and the depolarizing direction. More in general, due to its slow kinetics I_h_ is expected to selectively attenuate a neuron's response to synaptic input of low temporal frequency [41]. Since RBCs operate with graded potentials driven by changes in light intensity, an important factor in rod vision will be how RBCs respond to different temporal frequencies. We examined this in 17 RBCs by delivering, at different membrane potentials, small–amplitude sinusoidal current stimuli (50 s duration) modulated in frequency between 0.1 and 30 Hz (Figure 3B, top trace; details given in Materials and Methods). RBC membrane potential reacted with a sinusoidal trajectory, tapering in amplitude at the higher frequencies (Figure 3B, second trace from top). A second and more interesting behavior was observed in all RBCs, specifically when the membrane potential was in the range of activation of I_h_: taper occurred also at the low frequencies (Figure 3B, bottom two traces), resulting in a maximal response amplitude at an intermediate frequency. We computed, from each stimulus–response pair in all recorded cells, a complex input impedance (see Materials and Methods), which is a function of frequency. Examination of the modulus of the input impedance (for simplicity the *impedance profile*) confirmed that RBCs behave, to some degree, as band–pass filters (examples from several RBCs and membrane potentials are given in Figure 3C, thin noisy traces). This phenomenon may be quantified by a *band–pass index* (i_BP_), defined as the peak value of the impedance profile divided by its value at 0.1 Hz. The i_BP_ is unity for a low–pass impedance profile and takes increasing values the greater the band–pass character (Figure 3C, values indicated in graphs). Band–pass behavior was expressed by RBCs in two separate ranges of potentials: negative to −75 mV (Figure 3C a–c) and positive to −70 mV (Figure 3C e). Importantly, the former overlaps with the activation of I_h_, while the latter with that of the I_Kx_–like current. A comprehensive view over all 17 RBCs is given in a plot of i_BP_ versus membrane potential (Figure 3D, circles), which clearly shows the two ranges. The average resonant frequency—i.e. that of the impedance profile peak—differed significantly between the two ranges (Figure 3E, circles; 1.09±0.46 Hz at <−75 mV versus 0.50±0.20 Hz at >−70 mV, p<0.001 Wilcoxon–Mann–Whitney test).

### I_h_ is sufficient to explain the more negative range of RBC band–pass behavior

To strengthen the link between band–pass filtering and I_h_, we used a neuronal model incorporating a passive membrane, and I_h_ as the only voltage– and time–dependent conductance [31]. The model was adapted to each recorded RBC, by specifying leakage conductance and I_h_ with parameter values extracted from the voltage–clamp records (see Materials and Methods). The question we posed was whether this simplified forward model (i.e. with all parameters specified a priori) would correctly predict the experimental impedance profile for each cell and potential tested. Theoretical impedance profiles were obtained by linearizing the model at every chosen membrane potential (see Materials and Methods). As such, they are valid for small input signals (i.e. giving rise to small voltage fluctuations). In all RBCs in which both model and experimental profiles were available (*n* = 15), these were found to match very well at potentials more negative than about −70 mV (Figure 3C, red traces). The model confirmed that, when active, I_h_ attenuates frequencies below about 1 Hz and fully accounts for the band–pass response displayed by RBCs at potentials negative to −75 mV. Note that the steep drop in impedance at the higher frequencies is entirely expected and due to the cell's capacitance sitting in parallel with membrane conductances. The role of I_h_ at different membrane potentials is best appreciated by plotting the average i_BP_ and resonant frequency predicted by all cellular models (red curves in Figure 3D and 3E, respectively). Band–pass behavior is maximal at the I_h_ half–activation potential, as this is where its conductance is most sensitive to voltage changes. Interestingly, the resonant frequency is relatively stable over a wide range of potentials. In principle, the transient inward current (Figure 1C, arrow) could also contribute to RBC band–pass behavior [30]. What the simplified model clearly shows is that I_h_ alone can account for a large part of it. These graphs also point out that I_h_ cannot contribute to the frequency–tuning expressed by RBCs at potentials positive to −70 mV. This must instead rely upon other conductances such as the non–inactivating outward current (Figure 1D, traces with stars). Below we show that, after correcting for the artifactual membrane potential depolarization and increase in ohmic leakage introduced by patching on a small neuron, RBCs in darkness are estimated by the simplified model to be well in the range of I_h_ band–pass filtering.

### Blockade of I_h_ turns RBCs from band–pass to low–pass filters

**Figure 4:**
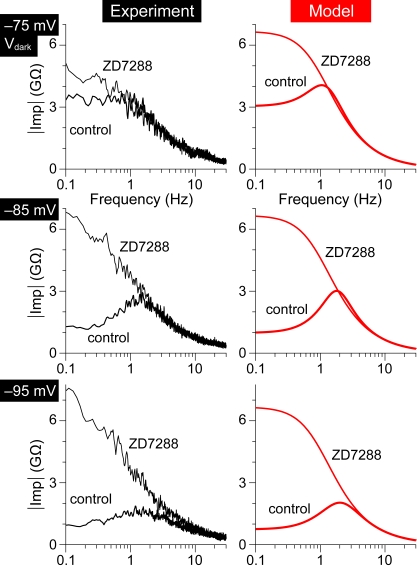
Blocking I_h_ converts the impedance profile of RBCs from band–pass to low–pass. *Left column*, Impedance profile of an RBC measured at V_dark_, V_dark_−10 mV and V_dark_−20 mV in control and during a nearly complete blockade of I_h_ with 1 µM ZD7288. At −85 mV and −95 mV ZD7288 greatly enhanced the cell's response to low–frequency input, converting its behavior from band–pass to low–pass one. This effect was less prominent but also present at the −75 mV (V_dark_). Impedance profiles obtained from average of 4–9 sinusoidal response sweeps. *Right column*, Impedance profiles predicted by a simplified model of the same RBC (see Figure 3C, legend) match rather well the experimental ones, except at −75 mV in ZD7288. The mismatch at this potential may be due to some activation of the current behind the upper range of band–pass behavior (cf. Figure 3D). ZD7288 was simulated by setting *G_h_* to zero. Detailed parameters used in modeling this cell were *G_h_* = 0.395 nS, *V_0.5_* = −90.5 mV, *k_m_* = 5.5 mV, *tau_max_* = 330 ms, *G_leak_* = 0.150 nS, *C* = 25 pF.

If I_h_ is responsible for the frequency tuning displayed by RBCs at potentials <−75 mV, a pharmacological blockade of the current should abolish it. We tested this by applying ZD7288 at 5 µM (*n* = 1) and 1 µM (*n* = 3). For each RBC its impedance profiles were determined, before and after addition of the drug to the bath, at the same set of potentials. This was achieved by injecting appropriate constant currents, onto which the sinusoidal stimuli were delivered. In all cases ZD7288 had a striking effect on the cells' impedance profiles, at membrane potentials within the activation range of I_h_ (Figure 4, left column). The impedance profiles at potentials negative to −75 mV were converted from band–pass (i_BP_ = 1.61±0.32) to low–pass ones (i_BP_ = 1.01±0.02; p<0.05 paired Wilcoxon–Mann–Whitney test) owing to an increase in the cell's response to low–frequency signals. The effect of ZD7288 on these RBCs was well predicted by their cellular models, in which G_h_ was set to zero (Figure 4, right column). No significant effect of ZD7288 was instead observed at potentials positive to −70 mV (not shown; i_BP_ = 1.37±0.44 in control versus i_BP_ = 1.34±0.46 in ZD7288; p = 0.63), thus confirming that a non HCN–mediated current is responsible for the band–pass behavior in that potential range. Over the course of these relatively long experiments, V_dark_ could typically fluctuate by several mV in either direction. No statistically significant effect of the blocker on V_dark_ could thus be established with such limited sample size.

### I_h_ quickens the response of RBCs to dim flashes of light

**Figure 5:**
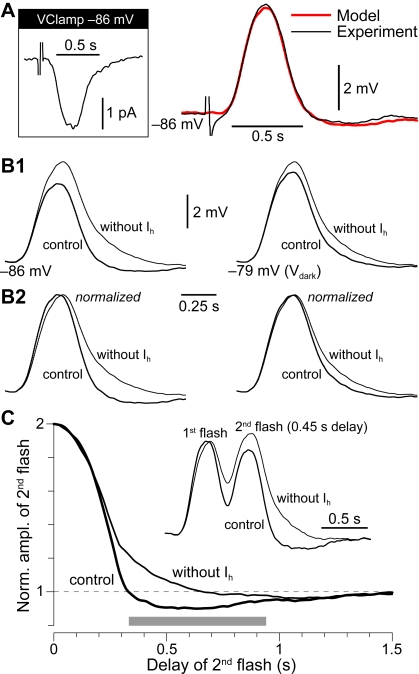
I_h_ quickens the dim flash response of RBCs and sharpens the detection of event coincidence. A, The band–pass filtering of RBCs operates not only on pipette–injected current, but also on light–evoked input. On the left, post–synaptic current (PSC) evoked by dim flash (3× threshold) recorded in voltage–clamp (−86 mV holding, average of 79). The same PSC was fed to the simplified model (see Figure 3C, legend) of the RBC under study (linearized at −84 mV, i_BP_ = 1.47). On the right (red trace), the predicted trajectory of post–synaptic potential (PSP) matches the one recorded in current–clamp with the same flash (black trace; membrane potential set at −86 mV by current injection, average of 61). B1, Flash–evoked PSPs predicted by the model at two different membrane potentials in control, and after removing I_h_ (by setting *G_h_* to 0). In the presence of I_h_, peak amplitude is reduced and the return to baseline is anticipated. B2, The latter remains true even after normalizing PSP amplitudes. Thus, the ‘price’ paid in terms of response amplitude reduction by expressing I_h_, is more than offset by a faster response of the RBC. C, Graph shows the amplitude of a second dim flash response following a first one with a brief delay (same flash strengths), as predicted by the model at −86 mV in control and after removing I_h_. Response amplitude is normalized to that of the same flash given in isolation. Inset shows the normalized voltage trajectories evoked by two flashes 0.45 s apart. For a duration of several hundred ms (indicated by a gray bar) I_h_ converts temporal summation of near–coincident flash responses (graph values>1) to attenuation (graph values<1).

How does the band–pass behavior described above affect the physiological input evoked by light? Being rod bipolars electrically compact [29], their response to small synaptic currents impinging at—or close to—V_dark_, should be predicted by the linearized cellular model with I_h_. This was verified by delivering a dim flash (∼1.5 photons/µm^2^·flash or 3 times the intensity eliciting about 50% response failures) while recording synaptic input currents in a voltage–clamped RBC (Figure 5A, left panel). The same flash was repeated in current–clamp to record the ensuing voltage–excursion shaped by the electrical properties of the RBC (Figure 5A, black trace in right panel). Note that at the holding potential (−86 mV in both V–C and C–C) I_h_ endowed the cell with a significant band–pass character (i_BP_ = 1.59). The synaptic current recorded in response to the flash was fed to the simplified model of the same RBC, and a theoretical voltage response was computed via its complex input impedance. The model's response (Figure 5A, red trace in right panel) matched the experimental one, confirming that RBCs react to small input signals in the same way, irrespective of whether they are synaptic or injected by a patch pipette. The role of RBC I_h_ in shaping flash–evoked potentials could, in principle, be obtained by pharmacological blockade. In practice, we found in pilot experiments that ZD7288 also influences presynaptic processing as apparent by complex changes in the synaptic currents evoked by the dimmest flashes. Albeit deserving future attention, this confounded the post–synaptic effect of the blocker. We instead exploited the predictive power of the simplified cellular model to look at the changes in the flash response caused by setting the I_h_ conductance to zero. In the absence of I_h_ the membrane potential transient becomes larger in amplitude and longer in duration (Figure 5B1). Note however that I_h_ does not perform a simple size scaling: normalizing amplitudes shows that I_h_ quickens RBC responses, mainly by accelerating their return to baseline (Figure 5B2). Thus, although band–pass filtering by I_h_ reduces dim flash response amplitude, this is more than compensated by a sharpening of its time course. An important consequence of this effect of I_h_ on individual responses will be a narrowing of the time window for the effective summation of two nearly–coincident input signals impinging on the same RBC. This is exemplified in Figure 5C by plotting the normalized response amplitude to a dim flash that follows a first one with a brief delay. For delays below about 300 ms responses summate to an amplitude greater than that of the same flash given in isolation (taken as the normalization factor). Beyond 300 ms the second response is actually attenuated. In the absence of I_h_ (*G_h_* set to zero in the model), summation extends to delays above 600 ms.

### I_h_ kinetics and flash–evoked input currents speed up at body temperature

**Figure 6:**
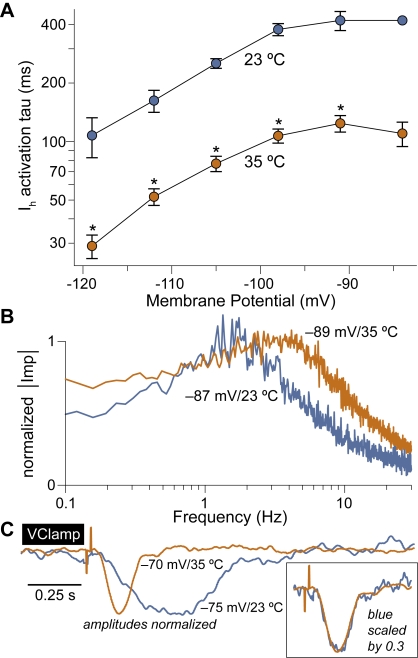
The effect of recording near body temperature checked in a limited number of RBCs. A, Voltage–dependent activation time constant of I_h_ near room temperature (23°C, blue circles) and body temperature (35°C, orange circles). I_h_ kinetics is markedly and significantly faster (* is p<0.05). Plot displays means and standard errors. B, The impedance profile of an RBC at 35°C (orange trace, −89 mV) displays a resonance peak shifted above 3 Hz. A representative profile from a cell at 23°C (similar potential) is superimposed for comparison (blue trace). Amplitudes are normalized. C, Average dim flash–evoked PSC recorded in two voltage–clamped RBCs, one at 35°C (orange trace, average of 25) and the other at 23°C (blue trace, average of 60). Amplitudes are normalized. The inset shows that the two PSC trajectories overlap once a time–wise scaling factor of 0.3 is applied to the blue trace.

We tested the effect on I_h_ kinetics, band–pass behavior, and flash responses, of recording in slices maintained near body temperature (∼35°C) instead of near room temperature (∼23°C) as in the above experiments. This was successful in a limited number of RBCs (*n* = 5), as under these conditions recordings were unstable and tended to be short–lived. I_h_ kinetics was found to become significantly faster (Figure 6A; p<0.05 Wilcoxon–Mann–Whitney test), with a maximum time constant *tau_max_* of 128±28 ms. I_h_ conductance at full activation *G_h_* was significantly larger (0.229±0.050 nS; p<0.05), whereas half activation potential *V_0.5_* and inverse slope factor *k_m_* were not found to differ (p = 0.8 and p = 0.4, respectively). Figure 6B (orange trace) illustrates a band–pass impedance profile from a RBC at 35°C. Note that the resonance peak is shifted to a higher frequency with respect to a representative RBC recorded at 23°C (blue trace; profiles are amplitude–normalized). Figure 6C compares the average dim flash–evoked input current in a RBC kept at 35°C (orange trace) with that from a representative RBC at 23°C (blue trace; amplitudes are normalized). These data indicate a generalized speeding up of the RBCs' input–output filtering characteristics, as well as of their light–evoked input signals, with temperature. It thus appears that our analysis of the role of I_h_ in RBCs at room temperature, may be extended to body temperature by a simple translation along the frequency scale.

### Effect of finite seal resistance on V_dark_ and i_BP_

Patch pipettes enable stable recordings, in the perforated or whole–cell configurations, from small neurons such as the rod bipolars of the mouse. Nonetheless, when target cells have input resistances in the GΩ–range, a significant measurement bias may be introduced by shunt through the pipette–membrane seal [32]. Although a proper estimate of seal resistance cannot realistically be obtained in RBCs (it would require simultaneous patching with two pipettes), this has been done in larger cells and found to vary widely over a range situated below 50 GΩ [42]. An imperfect seal, inserts in parallel to the neuronal membrane a shunt conductance to ground. This will depolarize the cell with respect to its unperturbed state [43]–[45]. The relevance of this issue to our work is twofold. First, a positive shift in V_dark_ will reduce the apparent role of I_h_ at physiological membrane potentials. Second, at any given potential within the range of I_h_ activation, the presence of the parasitic seal conductance will increase the ‘ohmic character’ of the cell and conversely diminish its band–pass behavior (cf. fig. 11D in [31]). In the following analysis we first model the currents that determine the apparent (i.e. measured) V_dark_ in a generic RBC with a patch–pipette sealed onto its membrane. We then proceed to predict the true (i.e. corrected) value of V_dark_ in an RBC having the average properties of our recorded population.

When the pipette is sealed to an RBC and held at its apparent V_dark_, the injected current is necessarily zero (steady–state conditions). Thus, if one assumes that at these negative potentials the only currents flowing through the cell's membrane are an ohmic leakage *I_leak_* and *I_h_* (see previous sections), these two must balance any current flowing through the seal:
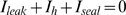
Expanding each term of this equation into the product of the underlying conductance and driving voltage, leads to
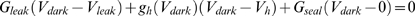
Note that the steady–state I_h_ conductance *g_h_(v)* is given by *eq. 2* (Materials and Methods), the seal conductance *G_seal_* has reversal potential zero (it is a shunt to ground), and V_dark_ is the apparent value.

**Figure 7:**
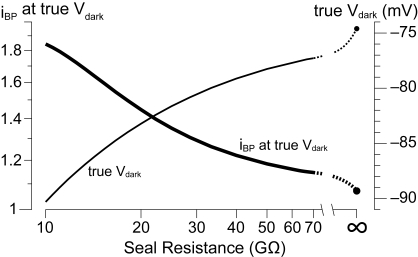
Correcting for the shunting effect of patch recording reveals the functional impact of I_h_ in unperturbed RBCs. The average dark membrane potential (V_dark_) observed in RBCs was −74.7 mV. If one assumes that during these recordings the pipette seal resistance was in a realistic range of a few tens of GΩ, the true (unperturbed) average value of V_dark_ may be predicted (see Results). For any given finite seal resistance, the true V_dark_ will be more negative than the recorded one, due to the depolarization introduced by the seal during the experiment (thin line, right axis). Only in the hypothetical case of an infinite seal resistance would the two values coincide (small circle at top right of thin line). The true V_dark_ and the simplified cellular model may be used to predict the band–pass index (i_BP_) of the average unperturbed RBC in darkness. Assuming for our experiments plausible seal resistances of less than 30–40 GΩ leads us to conclude that, at the true V_dark_ of RBCs, I_h_ is active and endows them with marked band–pass behavior (thick line, left axis).

Of the variables in *eq. 3* we know *V_h_* (−34 mV, Materials and Methods) and may also specify for *V_dark_* (−74.7 mV), *G_h_* (0.163 nS), *V_0.5_* (−91.4 mV) and *k_m_* (6.3 mV), the average values we found in our RBC recordings. Seal and membrane leak conductances sit in parallel and must appear lumped as their sum during an experiment, thus, for any arbitrary seal resistance (*1/G_seal_*) we can infer the true value of *G_leak_* to be 0.338 nS (the average *apparent* leakage in our recordings) minus *G_seal_*. At this point *eq. 3* may be solved for *V_leak_*, the reversal potential of the membrane leakage current. The unperturbed dark membrane potential of the average RBC (i.e. assuming a seal of resistance *1/G_seal_* was never made on it) can now be obtained by using *eq. 3* with *G_seal_* set to zero: *V_leak_* is now known and the equation is solved instead for a new value of *V_dark_*. This true dark membrane potential will necessarily be equal or negative to the recorded average of −74.7 mV. Figure 7 (thin curve) shows the true V_dark_ estimated assuming that a range of seal resistances were present in our recordings. While with a hypothetical perfect seal (*R_seal_* = ∞) the true V_dark_ would be equal to the average recorded one (Figure 7, top–right circle), with realistic seal resistances of a few tens of GΩ the difference between the two becomes very significant. More importantly, Figure 7 (thick curve) also shows that the band–pass behavior in darkness of the unperturbed average RBC, is predicted by the simplified cellular model to be much more robust if one again assumes a realistic range of seal resistance values.

### HCN1 and HCN2 have different expression patterns in the mouse retina

**Figure 8:**
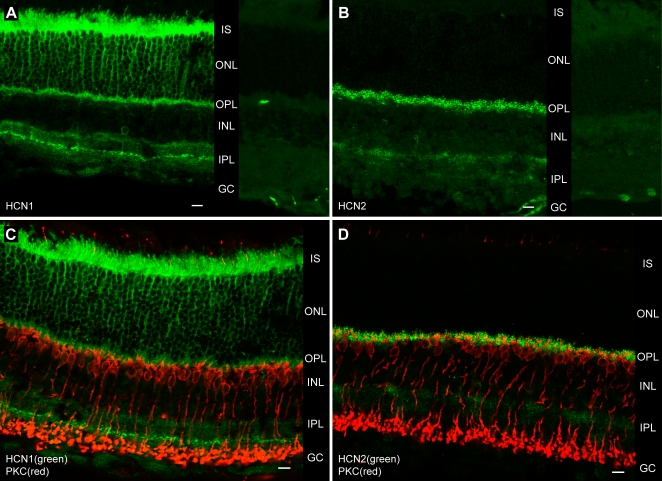
HCN1 and HCN2 channel isoforms localize differently in the mouse retina. A, Confocal micrograph of vertical frozen section through the retina treated with anti–HCN1 antibody and fluorescent secondary (left panel). Labeling is present in rod inner segments (IS), outer nuclear layer (ONL), outer (OPL) and inner plexiform layers (IPL). B, HCN2 are particularly evident in the OPL, but also weakly present in the external aspect of the IPL (left panel). Note that both the HCN1 and HCN2 stains were abolished by pre–incubation of the primary antibodies with their respective immunizing peptides (right panels). C, Double staining shows HCN1 subunits (green) together with RBCs labeled with mouse anti–PKC antibody (red). HCN1 do not seem to colocalize in any significant way with RBCs. D, The striking expression of HCN2 in small spots within the OPL (green), is strongly suggestive of a close association with the stubby dendrites of RBCs (red). Scale bars 10 µm.

A number of recent studies on rodents [19], [46], [47] suggest a segregation in the expression of subunit isoforms HCN1 and HCN2 between rod photoreceptors and RBCs. In an attempt to identify the HCN channel isoforms contributing to I_h_ in RBCs of the mouse retina, we examined this issue in detail by immunohistochemistry, using commercially available isoform–specific polyclonal antibodies. Immunofluorescence–stained vertical sections of the retina showed that HCN1 and HCN2 have clearly different distributions. HCN1 (Figure 8A, left panel) was strongly expressed in the rods' inner segments (IS), the outer nuclear layer (ONL), the outer plexiform layer (OPL) and at an intermediate level of the inner plexiform layer (IPL). Weaker expression was present diffusely throughout the IPL. HCN2 (Figure 8B, left panel) was instead primarily localized to the OPL with a dotted pattern of expression. HCN2 was also weakly present in the external half of the IPL. Sections treated with the two antibodies pre–incubated with their respective immunizing peptides, did not show any staining (Figure 8A/B, right panels) other than that of blood vessels (bright streaks), which is known to depend on an affinity of the secondary antibody. To further characterize the expression of these channel isoforms with respect to the first two elements of the primary rod pathway—rods and RBCs—we performed double stains using anti–PKC antibody, a marker of RBCs. The intense HCN1 expression within the IPL did not colocalize with the axons or synaptic terminals of RBCs (Figure 8C). The only possible site of significant HCN expression in RBCs was found to be their dendritic region, as both channel isoforms are present in the OPL. But while HCN1 was present diffusely throughout the OPL, HCN2 clearly appeared as beads closely associated with RBC dendrites (Figure 8D). This site was thus selected for a more detailed examination.

### HCN2 cluster in spots at the tip of RBC dendrites

**Figure 9:**
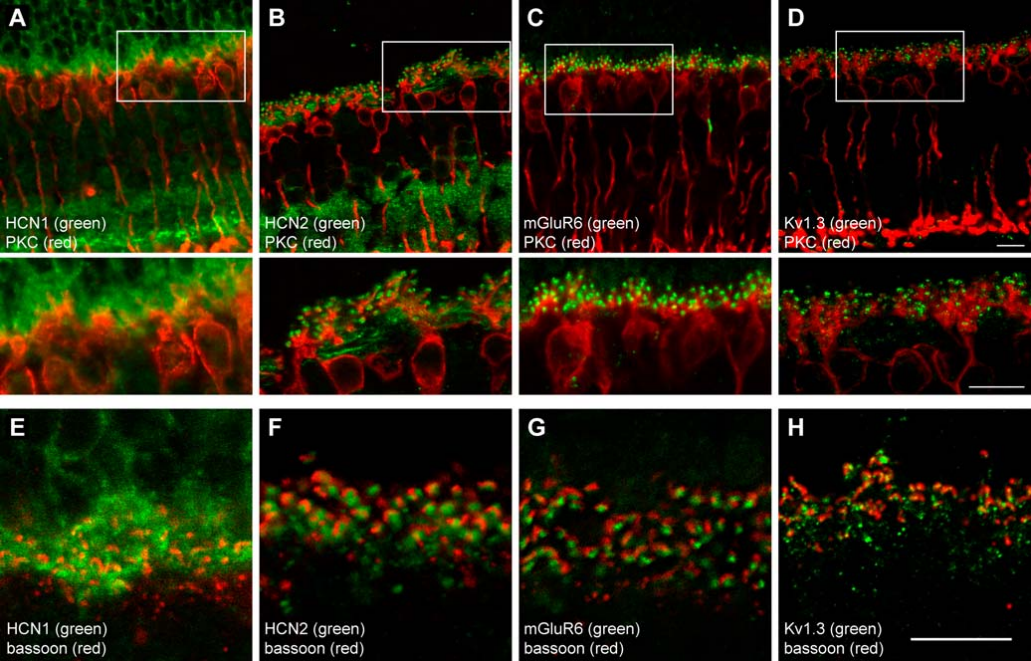
HCN2 concentrate at the tips of RBC dendrites, at sites of synaptic input from rods. A, Close–up view of the HCN1/PKC double staining. Further magnification of the field within the box is shown below. HCN1 express diffusely within the OPL, but do not colocalize with RBCs. B, Analogous close–up of an HCN2/PKC section shows that the channels' spotlike expression lines the tips of RBC dendrites. C, Double labeling with the postsynaptic receptor mGluR6 and PKC shows the same pattern observed with HCN2. D, Again, a similar arrangement is seen with the postsynaptically located *shaker* channel Kv1.3. E, Double labeling of HCN1 (green) and ribbon–contained Bassoon protein (red). HCN1 is clearly presynaptic. F, HCN2 (green) juxtapose with the arc–shaped ribbon complexes (red), in the same way as the postsynaptic mGluR6 (green in G) and Kv1.3 (green in H). Scale bars 10 µm.

Figure 9A and 9B present close–ups centered on the OPL, of the HCN1/PKC and HCN2/PKC double staining, respectively. The diffuse distribution of HCN1 without any obvious relationship to RBCs contrasts with that of HCN2. The latter distribution shows channels clearly organized in spots, lying at the tips of RBC dendrites (inset). This pattern of HCN2 expression was found to mimic that of the metabotropic glutamate receptor mGluR6 (Figure 9C), as well as the potassium channel subunit Kv1.3 (Figure 9D). Both are known to be located on the dendrites of RBCs at the sites of synaptic contact with rods [38], [48].

### HCN2 juxtapose with synaptic ribbons, like postsynaptic mGluR6 and Kv1.3

While this evidence points to a selective targeting of HCN2 in the immediate vicinity of the rod–RBC synapse, it does not clarify whether they are located pre– or postsynaptically. We addressed this issue by staining the presynaptic ribbon complex with an antibody against the cytomatrix protein Bassoon [49]. HCN1 were clearly and diffusely expressed by the rods up to the synaptic output region (Figure 9E). The HCN2 spots, on the other hand, were found to be juxtaposed to the rod synaptic ribbons (Figure 9F), again in complete similarity to the postsynaptic mGluR6s (Figure 9G) and Kv1.3s (Figure 9H). This set of immunohistochemical evidence thus suggests an expression of HCN2 channels on the dendrites of RBCs in coincidence with sites of synaptic input. This particular channel isoform could be partly or entirely responsible for the I_h_ current recorded in RBCs. Note that, because coincident on the same confocal plane, in principle one may not exclude that HCN2 labeling also affects horizontal cell terminals.

## Discussion

In this study we shed light on the functional implications of HCN expression in a non–spiking retinal neuron. Our data indicate a novel role for I_h_, in the early temporal processing of dim visual signals.

The I_h_ current expressed by mouse RBCs appears similar to that previously observed in voltage–clamp protocols in rat slices [19], [21], [50], although a detailed quantitative comparison is hampered by differences in the way the data are collected and presented. Also difficult is comparing I_h_ activation kinetics in RBCs (*tau_max_* 443 ms at room temperature) with the wide and partially overlapping ranges found in heterologous expression systems for HCN1 (*tau_max_* 170–750 ms) and HCN2 channels (*tau_max_* 0.5–7 s) [46], [51], [52]. While these data would tend to suggest HCN1 as the channels mediating I_h_ in RBCs, HCN2 expressed in native tissue are likely to be exposed to a number of modulatory influences, including auxiliary subunits, that may hasten its kinetics [53]. By performing a series of immunolabeling tests we found evidence in favor of a possible expression of HCN2 by RBCs, clustered at the tips of their short dendrites, in register with rod synaptic ribbons. While a general consensus exists on the distribution of HCN1, a previous study reported HCN2 expression mainly in RBC axonal synaptic boutons [19]. It should be pointed out however, that these results were obtained in rat and with an antibody targeting a different terminal of the HCN2 isoform. Moreover, a later study also on rat [22], showed a distribution pattern of HCN2 in the OPL similar to the one we describe here in mouse. In some experiments we dragged the recorded RBC back and forth by a short distance with the attached pipette, until the light response was lost. When this occurred, presumably due to mechanical lesion at the rod–RBC contact, I_h_ often disappeared as well. Later staining with LY confirmed that axon was still attached, thus bringing further support to a dendritic localization of the underlying HCN channels. Note that the possibility that HCN2 may be also expressed in horizontal cell axon terminals, where they invaginate within the rod spherules, cannot be ruled out.

A physiological involvement of I_h_ in the activity of a neuron depends critically on its membrane potential overlapping, at least transiently, the rather hyperpolarized range of I_h_ activation. This has been recognized as an open issue in I_h_–expressing bipolars [19], [50], essentially because the dark membrane potentials (V_dark_) reported so far, exhibited quite depolarized values (average in rat RBCs –45 mV [54]; in mouse RBCs –59 mV [55] and –60 mV [56]). Here we report instead a significantly more negative average value of V_dark_ in RBCs (–75 mV). We also show that, by taking into account the shunt unavoidingly introduced by the finite resistance of the pipette–membrane seal, the actual V_dark_ can be predicted to be even more negative in the unperturbed cell (previously recognized in retinal bipolar cells by [57]) and, importantly, well within I_h_–activation (Figure 7). In general, estimates of V_dark_ could be expected to be biased towards the depolarizing direction also because of tissue damage during slicing. For example, any rods that have lost their outer segment will hyperpolarize, providing tonic depolarization to their postsynaptic RBCs. In support of this, in the early phase of the project we experienced, experiment by experiment, a progressive shift in V_dark_ from –50/–60 to –70/–80 mV, which paralleled improvements in the appearance of the outer retina and most importantly a great increase in the occurrence of light–responding RBCs. Such more negative V_dark_ values seem entirely reasonable, once the following additional points are considered. The K^+^ equilibrium potential is around –98 mV (23°C) in standard extracellular AMES medium, and in the same experiments presented here we found the low–impedance Müller glia to rest concordantly between –95 and –99 mV. Importantly, LVA calcium channels at the RBC output synapse have been shown able to mediate glutamate exocytosis, when the membrane potential is sufficiently negative to relieve their inactivation [35], [36]. *In vivo* ERG recordings documented the impact that I_h_ inhibition has on the component of the outer retina's response that reflects RBC activity [14].

When and why would RBCs require the frequency tuning introduced by I_h_? It is now well established that band–pass filtering is already present upstream, in rods. Here, an I_Kx_ current with slow–feedback properties analogous to those of I_h_, but operating over the more depolarized range of potentials of photoreceptors, shapes dim light signals in a way similar to what we demonstrate here I_h_ does in RBCs [24], [37]. In rods I_h_ comes into play at higher light intensities [24] and one may wonder if its functional counterpart in RBCs could be the slow–activating outward current (Figure 1D), which is conceivably mediating the band–pass behavior we observed positive to –70 mV (Figure 3D). A specular role of I_h_ and I_Kx_ in rods and RBCs would be an elegant arrangement to match their differing dark membrane potentials and opposite light–response polarities. In addition to ion channels, synaptic transmission [58] and amacrine feedback [59] may contribute band–pass filtering in the early rod visual system. It thus appears that active suppression of low temporal frequencies is an important process here, distributed at least along the first stages of signal convergence in the retina, and perhaps up to the ganglion cells [20], [60]. One can think of at least two reasons for its existence. One would be to preserve the high temporal frequency content of light input [25]. Phototransduction in the rod outer segment is inherently slow, and further electrotonic spreading of the ensuing signal could occur as it proceeds in a graded manner to the ganglion cells. Filtering may thus operate, in tandem with amplifying mechanisms (e.g. synaptic transfer gain, network convergence), to counteract this loss of information. Moreover, slow changes in background light are probably of scarce perceptual relevance to the animal. The other reason would be to improve the signal–to–noise ratio [61], with noise arising in the retina from a number of different sources [62]. Band–pass filtering, by restricting the time window for temporal summation (Figure 5C), could sharpen coincidence detection in conjunction with a thresholding output synapse such as that made by rods [63], [64], and thereby help reject uncorrelated spontaneous photoisomerizations. Particular interest in understanding the role of HCN channels in retinal function has been spurred by the visual side–effects in cardiac patients treated with I_h_ inhibitors (reviewed by [16]). Symptoms prevail in darkness or dim light, and include phosphenes (flashes of light) and stroboscopic or blurred vision. The contribution of I_h_ to band–pass filtering and the possible functional implications of the latter in rod vision, discussed above, may clearly account for some or all of these symptoms. Given the high degree of convergence in the rod pathway, testing these hypotheses may require recording downstream of RBCs.

HCN channels are widely expressed in the central nervous system, and their best described function is probably the contribution they give to neuronal pacemaking and network rhythmicity [2], [65]. Of greater relevance in this context is their action in high–pass filtering subthreshold synaptic input in hippocampal [13], [66] and other cortical pyramidal neurons [7], [67]. Here, the primary outcome of this function may be to regulate the integration of input impinging on proximal and distal sites of electrically extended dendrites [68], [69]. In retinal RBCs instead, the channels that mediate the I_h_ current, possibly HCN2, appear ideally suited to sharpen dim light responses, because their relatively slow activation/deactivation kinetics are close to that of the sensory transductive element—the photoreceptor outer segment. In many systems the half–activation potential of the different HCN isoforms is shifted by rising cAMP levels [2], [10], but other influences have also been discovered [70], [71]. Intraretinal modulatory systems (e.g. dopaminergic amacrines), could thus influence the state of these channels. The apparent clustering of HCN2 channels on the dendrites of RBCs, at points of synaptic input, is puzzling. In such a seemingly isopotential neuron [29], channel localization should not matter for electrophysiological function, and the HCN2 might as well have been uniformly distributed over the cell's surface. This raises the intriguing possibility of some direct interaction between the HCN2 and the post–synaptic machinery. Channel modulation could perhaps be taking place in response to changes in ambient light [72], for example extending out of the deep scotopic range, the temporal filtering demonstrated in this study.
